# Promotion of shade avoidance by BBX5 involves activation of *PIF4* along with auxin biosynthetic and signaling genes

**DOI:** 10.1371/journal.pgen.1012177

**Published:** 2026-06-05

**Authors:** Fengyue Zhao, Rongbo Yang, Zhaoqing Song, Yeting Bian, Yuntao Xiao, Dongqing Xu

**Affiliations:** State Key Laboratory of Crop Genetics & Germplasm Enhancement and Utilization, National Center for Soybean Improvement, College of Agriculture, Nanjing Agricultural University, Nanjing, China; University of Kentucky, UNITED STATES OF AMERICA

## Abstract

Neighbor proximity triggers changes in light quality that regulate various developmental and physiological processes in plants. phytochrome B (phyB)-PHYTOCHROMEINTERACTING FACTOR 4 (PIF4) module serves as a central regulatory hub enabling plants to accurately perceive and respond to shade cues. Here, we identify B-box PROTEIN 5 (BBX5) as a positive regulator of shade avoidance. phyB interacts with BBX5 and promotes its protein stability. Conversely, the E3 ubiquitin ligase CONSTITUTIVELY PHOTOMORPHOGENICLY 1 (COP1) associates with and destabilizes BBX5 via the 26S proteasome system in shade. BBX5 binds to the *PIF4* promoter to upregulate its expression during the early phase of shade exposure, and directly associates with the promoters of auxin biosynthetic and signaling genes *YUCCA8* (*YUC8*) and *INDOLE-3-ACETIC ACID INDUCIBLE 19* (*IAA19*) to activate their expression in shade. Our study reveals that BBX5 acts as a transcriptional activator of *PIF4*, *YUC8* and *IAA19* to promote plant growth and development in response to shade signals.

## Author summary

BBX5 is essential for flowering-time regulation, however, its involvement in shade-avoidance responses remains elusive. Here, we demonstrate that BBX5 acts as a positive regulator of shade avoidance. The stability of the BBX5 protein is precisely modulated by phyB and COP1 in opposing manners. Mechanistically, BBX5 promotes the transcription of *PIF4* and auxin biosynthetic and responsive genes to promote growth under shade conditions. Our study reveals a molecular framework wherein BBX5 orchestrates shade avoidance responses.

## Introduction

Light is a key environmental factor regulating plant growth and development [1]. As sessile organisms, sun-loving plants grown in high planting density or under canopy conditions display elongated hypocotyls and petioles, enhanced leaf hyponasty, reduced branching, diminished leaf expansion, and accelerated flowering and senescence, collectively termed shade avoidance syndrome (SAS) [2,3].

Sunlight comprises a spectrum of wavelengths, including ultraviolet-B (UV-B; 280–320 nm), blue (B; 400–500 nm), red (R; 600–700 nm), and far-red (FR; 700–750 nm) light. Chlorophylls and carotenoids within plant cells primarily absorb B and R light for photosynthesis, whereas FR light is largely transmitted or reflected by the canopy. This results in a dramatic reduction in the R/FR ratio [4–6]. Perception of low R/FR by shade-intolerant plants triggers SAS, enabling them to compete for limited light [6,7].

phytochromes (phys) are photoreceptors for R and FR light in plants [8,9]. They are maintained in two photoconvertible isoforms within plant cells. The biologically active Pfr form absorbs FR, while the biologically inactive form perceives R. The Pr form is localized in the cytoplasm under the dark conditions. Upon R light irradiation, the Pr form is converted into Pfr, which subsequently translocates into the nucleus. The Pfr form absorbs FR and reverts to the inactive Pr ground state [10,11]. Thus, the steady-state ratio of R/FR determines the Pfr to Pr equilibrium, thereby governing SAS in plants. Among the five phys (phyA-E), phyB plays a dominant role in mediating SAS triggered by low R/FR [12,13]. The FR-absorbing photoreceptor phyA accumulates to prevent excessive SAS under prolonged deep shade [14,15].

The Pfr form phyB interacts with a subgroup of bHLH-type transcription factors, the PHYTOCHEME INTERACTING FACTORs (PIFs: PIF1, 3, 4, 5 and 7). These molecular events trigger rapid phosphorylation, ubiquitination, and degradation of PIF1, 3, 4 and 5, as well as phosphorylation of PIF7 [10,11]. Low R/FR inactivates phyB, leading to accumulation of PIF4 and PIF5, and promoting dephosphorylation and phase separation of PIF7 [16–18]. PIF4, PIF5 and PIF7 directly bind to the promoters of numerous auxin biosynthetic and signaling genes to activate their transcription, and thereby promoting plant growth and development [16,17,19–21].

B-box domain containing proteins (BBXs) are a family of transcription factors and/or regulators that regulate multiple light-controlled developmental processes in plants [22–24]. A subset of BBX proteins integrate shade signals, circadian clock cues and various hormonal signaling pathways to regulate SAS [25,26]. BBX7 and BBX8 directly bind to the promoters of *CIRCADIAN CLOCK ASSOCIATED 1* (*CCA1*) and *LATE ELONGATED HYPOCOTYL* (*LHY*) to activate their transcription specifically during the early morning. CCA1 and LHY up-regulate the expression of *PIF4*, and hence leading to accumulation of PIF4 abundance to promote shade-triggered hypocotyl growth [27]. BBX21 inhibits SAS by activating the transcription of numerous genes involved in phyB, auxin, and brassinosteroid (BR) signaling [28,29]. BBX24 integrates jasmonic acid (JA) and gibberellin (GA) signaling to promote SAS [30,31]. In addition, BBX24, BBX25, and BBX28 promote SAS through COP1-mediated signaling pathway [30,32]. These findings indicate that BBXs regulate SAS through complex pathways integrating diverse external and internal signals.

In this study, we report that BBX5 acts as positive regulator of SAS in *Arabidopsis*. Loss of BBX5 function resulted in elongated hypocotyls and petioles, whereas transgenic plants overexpressing BBX5 exhibited shortened hypocotyls and petioles in shade. Photoreceptor phyB directly interacted with and stabilized BBX5, whereas shade signals interfered with this protein-protein interaction. The E3 ubiquitin ligase COP1 bound to the Valine-Proline (VP) motif located in the middle potion of BBX5 and promoted its degradation via the 26S proteasome system in shade. On the one hand, BBX5 associated with the promoter regions of *PIF4* to upregulate its transcription, thereby elevating PIF4 protein levels. On the other hand, BBX5 directly binds to the promoters of auxin biosynthetic gene *YUC8* and the auxin signaling gene *IAA19*, activating their transcription in shade. In summary, our study provides novel insights into the complex molecular network underlying plant shade responses.

## Results

### BBX5 promotes shade-induced hypocotyl and petiole elongation

**Fig 1:**
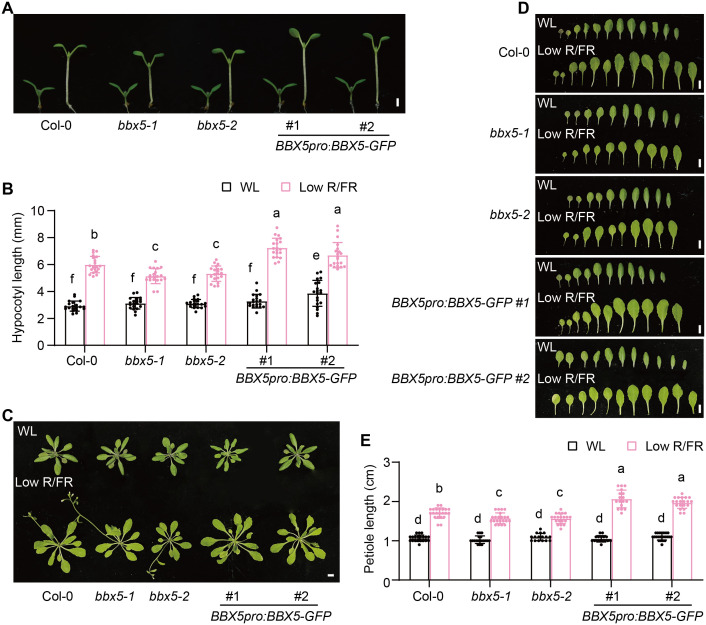
BBX5 promotes hypocotyl and petiole growth in low R/FR. **(A-B)** Hypocotyl phenotypes (**A**) and length (**B**) of Col-0, *bbx5-1*, *bbx5-2*, *BBX5pro:BBX5-GFP #1* and *BBX5pro:BBX5-GFP #2* seedlings. Plants were grown in WL for 3 d, then either kept in WL or transferred to low R/FR conditions for an additional 3 **d.** Scale bar = 1 mm. Values are means ± SE (n ≥ 20). Letters above the bars indicate significant differences (*P* < 0.05), as determined by one-way ANOVA with Tukey’s post hoc analysis. **(C)** Phenotypes of 30 d-old Col-0, *bbx5-1*, *bbx5-2*, *BBX5pro:BBX5-GFP #1* and *BBX5pro:BBX5-GFP #2* seedlings grown under WL or low R/FR conditions. **(D)** Petiole phenotypes of 30 d-old Col-0, *bbx5-1*, *bbx5-2*, *BBX5pro:BBX5-GFP #1* and *BBX5pro:BBX5-GFP #2* seedlings ‌‌grown under WL or low R/FR conditions. Scale bar = 1 cm. **(E)** Petiole length of the fifth leaf from plants analyzed in (**C)** and **(D)**. Values are means ± SE (n ≥ 20). Letters above the bars indicate significant differences (*P* < 0.05), as determined by one-way ANOVA with Tukey’s post hoc analysis. In panels **(C-E)**, plants were grown in WL for 20 d, then either kept in WL or transferred to low R/FR for an additional 10 d.

BBX proteins play critical roles in various light-dependent developmental processes in plants [22,24]. To investigate the biological function of BBX5, we generated *bbx5* mutants (*bbx5–1* and *bbx5–2*) using CRISPR-Cas9 techniques, and also produced two independent transgenic lines over-expressing C-terminally GFP-tagged *BBX5* driven by its native promoter (*BBX5pro:BBX5-GFP #1* and *#2*) (S1 Fig). The Col-0 (wild-type, WT), *bbx5–1, bbx5–2* and *BBX5pro:BBX5-GFP #1* seedlings showed comparable hypocotyl lengths, while the hypocotyl length of *BBX5pro:BBX5-GFP #2* was slightly longer than WT in white light (WL) (Fig 1A-B). *bbx5–1* and *bbx5–2* seedlings exhibited shortened hypocotyls, whereas *BBX5pro:BBX5-GFP #1* and #2 transgenic seedlings displayed elongated hypocotyls when they were grown in low R/FR (Fig 1A-B). *bbx5* mutants and transgenic plants overexpressing *BBX5* displayed petiole lengths comparable to WT in WL. However, the petiole length of *bbx5–1* and *bbx5–2* was significantly shorter than that of WT, whereas overexpression of *BBX5* led to elongated petioles grown in low R/FR (Fig 1C-E). Together, these results suggest that BBX5 functions as a positive regulator of shade-induced hypocotyl and petiole elongation in *Arabidopsis*.

Next, we examined the expression patterns of BBX5 at both transcriptional and protein levels in response to WL or shade. The transcription of *BBX5* was upregulated in etiolated Col-0 seedlings upon transferred to WL 1 and 3 h, and subsequently returned to basal levels by 6, 12 and 24 h (S2A Fig). The transcript levels of *BBX5* remained stable in Col-0 seedlings grown in WL upon transferred to low R/FR at 1, 3 and 6 h, but decreased at 12 and 24 h. Under continuous WL or low R:FR conditions, *BBX5* expression showed no significant changes (S2B Fig). These results indicate that *BBX5* is differentially regulated at the transcriptional level by WL and low R/FR signals. The BBX5 protein levels were gradually increased in *BBX5pro:BBX5-GFP* #1 grown in darkness upon transferred to WL for 1, 3 and 6 h, but decreased when kept in WL for 12 and 24 h (S2C Fig). These results suggest that light induces the accumulation of BBX5. BBX5-GFP protein levels were clearly reduced in WL-grown *BBX5pro:BBX5-GFP* #1 seedlings after low R/FR exposure for 1, 3 and 6 h, but subsequently accumulated after 12 and 24 h of low R/FR exposure or under continuous low R/FR conditions (S2D Fig). These results indicate that BBX5 protein levels decrease during early shade exposure but re-accumulate after prolonged shading in *Arabidopsis.*

### Shade disrupts the interaction between phyB and BBX5

**Fig 2:**
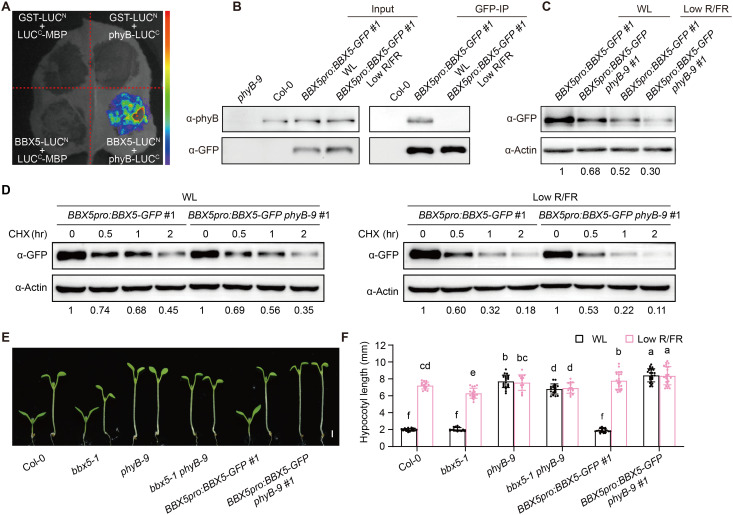
phyB interacts with and stabilizes BBX5. **(A)** Luciferase complementation imaging (LCI) assays showing BBX5 interacted with phyB in *Nicotiana benthamiana* leaves. Full‐length phyB and BBX5 were fused to the split N‐ or C‐terminal (LUC^N^ or LU^CC^) fragments of LUC respectively. MBP-LUC^N^ and LU^CC^-GST were used as negative controls. **(B)** Co-immunoprecipitation (Co-IP) analysis shows that BBX5 preferentially interacted with Pfr form of phyB *in vivo*. Col-0 and *BBX5pro: BBX5-GFP #1* seedling were grown in darkness for 4 d, then either kept in WL or transferred to low R/FR for an additional 1 h respectively. The total proteins were extracted and treated with WL or low R/FR for an additional 5 min and then incubated with anti-GFP magarose beads. The total and precipitated proteins were subjected to immunoblot analysis using antibodies against phyB and GFP respectively. *phyB-9* was used as the negative control. **(C)** Immunoblots analysis showing BBX5-GFP protein levels in *BBX5pro: BBX5-GFP #1* and *BBX5pro: BBX5-GFP phyB-9 #1* seedlings. Plants were grown in WL for 5 d, then either kept in WL or transferred to low R/FR for an additional 1 h respectively. Actin was used as the loading control. Numbers below the immunoblots indicate the relative intensities of BBX5-GFP bands normalized to those of loading controls, and the ratio was set to 1 for the first lane of each group. **(D)** Immunoblots analysis showing BBX5-GFP protein levels in *BBX5pro: BBX5-GFP #1* and *BBX5pro: BBX5-GFP phyB-9 #1* seedlings after CHX treatment. Plants were grown in WL for 5 d, then treated with CHX (500 μM) for the indicated times under WL or low R/FR conditions respectively. Actin was used as the loading control. Numbers below the immunoblots indicate the relative intensities of BBX5-GFP bands normalized to those of loading controls, and the ratio was set to 1 for the first lane of each seedling. **(E-F)** Hypocotyl phenotypes (**E**) and length (**F**) of Col-0, *bbx5–1*, *phyB-9*, *bbx5–1 phyB-9*, *BBX5pro:BBX5-GFP #1* and *BBX5pro:BBX5-GFP phyB-9 #1* seedlings. Plants were grown in WL for 3 d, then either kept in WL or transferred to low R/FR for 3 d. Scale bar = 1 mm. Values are means ± SE (n ≥ 20). Letters above the bars indicate significant differences (*P* < 0.05), as determined by one-way ANOVA with Tukey’s post hoc analysis.

phyB senses low R/FR signals and thus initiates SAS in plants [13]. We therefore tested whether phyB interacts with BBX5. Luciferase complementation imaging (LCI) assays revealed that LUC signals were clearly observed in *Nicotiana benthamiana* leaves when BBX5-LUC^N^ and LU^CC^-phyB were transiently co-expressed (Fig 2A). The respective negative controls did not produce any detectable LUC signals (Fig 2A). To verify these results, we performed co-immunoprecipitation (Co-IP) assays using *BBX5pro:BBX5-GFP #1* transgenic line and endogenous phyB antibodies. As shown in Fig 2B, BBX5-GFP co-immunoprecipitated with phyB in *BBX5pro:BBX5-GFP #1* seedlings grown in WL*,* but not in low R/FR, suggesting that shade impairs the association of phyB with BBX5. Considering that low R/FR triggers the conversion of a large portion of phyB from the Pfr to the Pr form, which rapidly shifts to the cytoplasm [13], these results imply that BBX5 preferentially associates with biologically active Pfr form in *Arabidopsis*.

phyB controls the protein levels of a set of its interacting partners [10]. We therefore examined whether phyB regulates the accumulation of BBX5 protein. The abundance of BBX5-GFP in *BBX5pro:BBX5-GFP phyB-9* #1 was markedly reduced compared with that in *BBX5pro:BBX5-GFP* #1 grown under WL, low R/FR or R light conditions (Figs 2C and S3), suggesting that phyB stabilizes the BBX5 at the protein level. The transcript levels of *BBX5* were not significantly altered in Col-0, *phyB-9* and *phyB-CFP* (*PBC*) seedlings grown in WL and low R/FR. It was significantly increased in *phyB-9* seedlings grown in R light (S4A Fig). In addition, the transcript levels of *BBX5* in *BBX5pro:BBX5-GFP phyB-9* #1 were slightly elevated compared to those in *BBX5pro:BBX5-GFP* #1 when they were grown in WL, low R/FR or R light conditions (S4B Fig). These results suggest that phyB represses *BBX5* at the transcriptional level. To further validate that phyB stabilizes the BBX5, we compared the BBX5-GFP protein levels in *BBX5pro:BBX5-GFP* #1 and *BBX5pro:BBX5-GFP phyB-9* #1 seedlings treated with cycloheximide (CHX), a protein synthesis inhibitor. BBX5-GFP proteins degraded more rapidly in the *BBX5pro:BBX5-GFP phyB-9* #1 than in *BBX5pro:BBX5-GFP* #1 under both WL and low R/FR conditions (Fig 2D), indicating that phyB is required for BBX5 stabilization. In addition, BBX5-GFP protein levels remained stable in both *BBX5pro:BBX5-GFP* #1 and *BBX5pro:BBX5-GFP phyB-9* #1 seedlings upon simultaneous treatment with MG132 and CHX (S5 Fig). These results indicate that phyB enhances BBX5 protein stability by repressing its 26S proteasome-dependent degradation.

Next, we investigated the genetic relationship between *phyB* and *BBX5*. *phyB-9* displayed dramatically elongated hypocotyls in WL or low R/FR (Fig 2E-F), consistent with previous studies [13]. The hypocotyl length of *bbx5–1* and *BBX5pro:BBX5-GFP* #1 was significantly shorter than that of *phyB-9* grown in WL. The *bbx5–1* seedlings were shorter than *phyB-9,* while the hypocotyl length of *BBX5pro:BBX5-GFP* #1 was comparable to that of *phyB-9* when they were grown in low R/FR (Fig 2E-F). *phyB-9 bbx5–1* were slightly shorter than *phyB-9,* whereas *BBX5pro:BBX5-GFP phyB-9* #1 seedlings were longer than *phyB-9* when grown in WL or low R/FR (Fig 2E-F). Together, these genetic results suggest that phyB and BBX5 likely act interdependently in regulating SAS in *Arabidopsis*.

### COP1 interacts with and de-stabilizes BBX5

**Fig 3:**
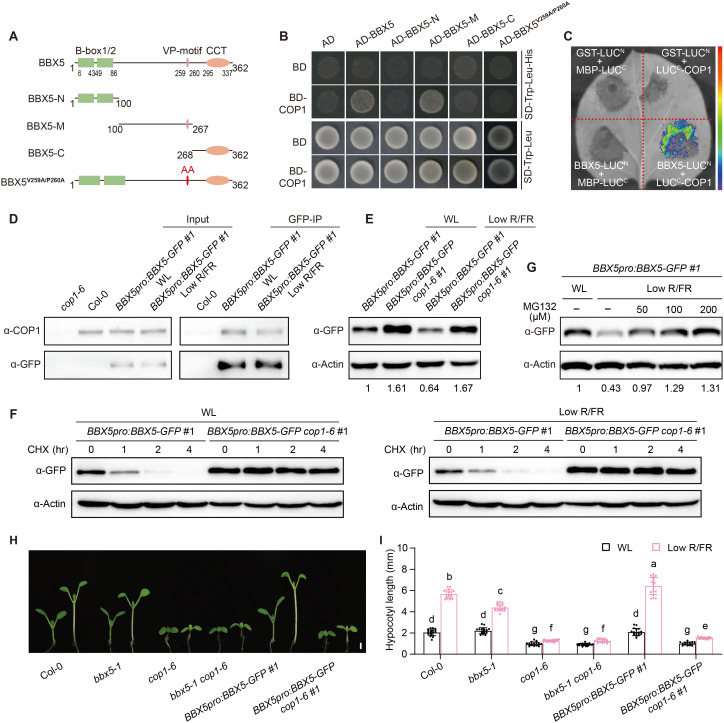
COP1 interacts with and de-stabilizes BBX5 via the 26S proteasome system. **(A)** Protein structures of full-length, truncated and mutated BBX5 used in the yeast two‐hybrid assays. **(B)** Yeast two-hybrid assays showing the interaction of BBX5 with COP1. **(C)** Luciferase complementation imaging (LCI) assays showing BBX5 interacted with COP1 in *Nicotiana benthamiana* leaves. Full‐length BBX5 and COP1 were fused to the split N‐ or C‐terminal (LUC^N^ or LU^CC^) fragments of LUC respectively. MBP-LUC^N^ and LU^CC^-GST were used as negative controls. **(D)** Co-immunoprecipitation (Co-IP) analysis showing that BBX5 interacted with COP1 *in vivo*. Col-0 and *BBX5pro: BBX5-GFP #1* seedling were grown in darkness for 4 d, then either kept in WL or transferred to low R/FR for an additional 1 h respectively. The total proteins were extracted and treated with WL or low R/FR for an additional 5 min and then incubated with anti-GFP magarose beads. The total and precipitated proteins were subjected to immunoblot analyses using antibodies against COP1 and GFP respectively. *cop1-6* was used as the negative control. **(E)** Immunoblots analysis showing BBX5-GFP protein levels in *BBX5pro: BBX5-GFP #1* and *BBX5pro: BBX5-GFP cop1-6 #1* seedlings. Plants were grown in WL for 5 d, then either kept in WL or transferred to low R/FR for an additional 1 h respectively. Actin was used as the loading control. Numbers below the immunoblots indicate the relative intensities of BBX5-GFP bands normalized to those of loading controls, and the ratio was set to 1 for the first lane of each group. **(F)** Immunoblots analysis showing BBX5-GFP protein levels in *BBX5pro: BBX5-GFP #1* and *BBX5pro: BBX5-GFP cop1-6 #1* seedlings after CHX treatment. Plants were grown in WL for 5 d, then treated with CHX (500 μM) for the indicated times under WL or low R/FR conditions respectively. Actin was used as the loading control. Numbers below the immunoblots indicate the relative intensities of BBX5-GFP bands normalized to those of loading controls, and the ratio was set to 1 for the first lane of each seedling. **(G)** Immunoblot analysis showing BBX5-GFP protein levels in *BBX5pro: BBX5-GFP #1* seedlings. Plants were grown in WL for 5 d, then treated with various concentrations of MG132 (50, 100, or 200 μM) for 3 h, and subsequently transferred to low R/FR for 1 **h.** Actin was used as the loading control. Numbers below the immunoblots indicate the relative intensities of BBX5-GFP bands normalized to those of loading controls, and the ratio was set to 1 for the first lane of each group. **(H-I)** Hypocotyl phenotypes and length of Col-0, *bbx5-1*, *cop1-6*, *bbx5-1 cop1-6*, *BBX5pro:BBX5-GFP #1* and *BBX5pro:BBX5-GFP cop1-6 #1* seedlings. Plants were grown in WL for 3 d, then either kept in WL or transferred to low R/FR conditions for an additional 3 **d.** Scale bar = 1 mm. Values are means ± SE (n ≥ 20). Letters above the bars indicate significant differences (*P* < 0.05), as determined by one-way ANOVA with Tukey’s post hoc analysis.

The E3 ubiquitin ligase COP1 targets a set of BBXs for ubiquitination and degradation [22,24]. We thus performed yeast two-hybrid assays to test whether COP1 interacts with BBX5. As shown in Fig 3A-B, COP1 interacted with BBX5 in yeast cells. To map the region of BBX5 responsible for its interaction with COP1, we divided BBX5 into three portions: BBX5-N (1–100) containing two conserved B-box domains, BBX5-M (100–267) possessing a Valine-Proline (VP)-motif and BBX5-C carrying a CCT domain (Fig 3A). COP1 interacted with BBX5-M, but not with BBX5-N or BBX5-C. BBX5-M contains a VP-motif, which is a potential COP1-interacting site [33]. COP1 did not interact with BBX5^VP/AA^, in which the VP-motif was mutated to AA (Fig 3A-B). These results suggest that COP1 associates with the VP-motif of BBX5. Furthermore, LUC signals were detected in *Nicotiana benthamiana* leaves when BBX5-LUC^N^ and LU^CC^-COP1 were transiently co-expressed (Fig 3C). The respective negative pairs did not produce any detectable LUC signals. Co-IP assays showed that BBX5-GFP co-immunoprecipitated with COP1 in *BBX5pro:BBX5-GFP* #1 seedlings grown under WL or low R/FR conditions (Fig 3D). Together, these results indicate that COP1 interacts with BBX5 in *Arabidopsis*.

Next, we examined whether COP1 affects the BBX5 abundance. *BBX5pro:BBX5-GFP cop1–6 #1* seedlings accumulated slightly higher levels of BBX5-GFP protein compared with those in *BBX5pro:BBX5-GFP #1* grown in WL (Fig 3E). BBX5-GFP protein levels were lower in *BBX5pro:BBX5-GFP #1* grown in low R/FR compared with those in WL. BBX5-GFP obviously accumulated in *BBX5pro:BBX5-GFP cop1–6 #1* compared with those in *BBX5pro:BBX5-GFP #1* grown in low R/FR or darkness (Figs 3E and S6)*.* The transcript levels of *BBX5* were not significantly altered in *BBX5pro:BBX5-GFP #1* and *BBX5pro:BBX5-GFP cop1–6 #1* seedlings grown in darkness, WL or low R/FR conditions, suggesting that COP1 does not regulate the transcription of *BBX5* (S7 Fig)*. BBX5-GFP* proteins degraded in *BBX5pro:BBX5-GFP* #1 but remained stable in *BBX5pro:BBX5-GFP cop1–6* #1 when treated with the protein synthesis inhibitor CHX under both WL and low R/FR conditions (Fig 3F). Together, these results suggest that COP1 promotes the degradation of BBX5 in *Arabidopsis*. The decrease of BBX5-GFP protein levels in *BBX5pro:BBX5-GFP #1* upon low R/FR exposure was markedly inhibited by treatment with 50 μM MG132 (a proteasome inhibitor), and this inhibition became more evident when the concentration of MG132 was increased to 100 and 200 μM (Fig 3G). Although the hypocotyl length in *bbx5–1 cop1–6* was comparable to that of *cop1–6*, *BBX5pro:BBX5-GFP cop1–6 #1* were significantly longer than *cop1–6* in low R/FR (Fig 3H-I), indicating that the function of BBX5 in shade requires a functional COP1*.* Together, these results suggest that COP1 promotes the degradation of BBX5 via the 26S proteasome system in plant cells.

### BBX5 upregulates the expression of *PIF4* during the early phase of shade exposure

**Fig 4:**
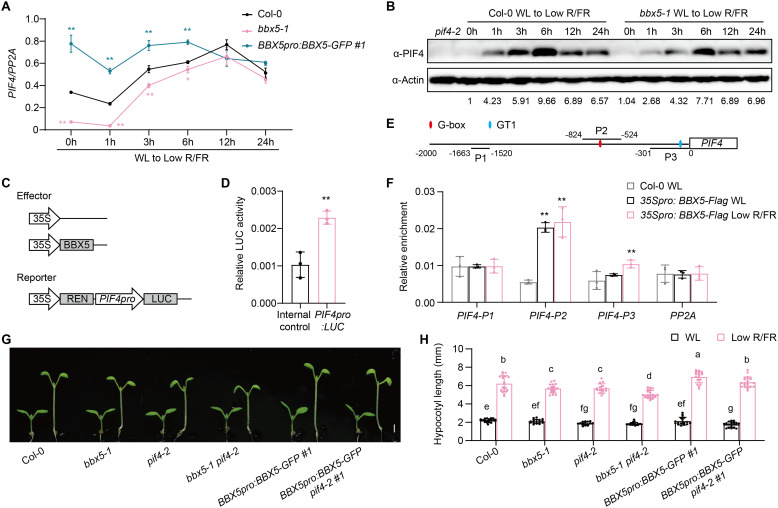
BBX5 upregulates the transcription of *PIF4* in early shade. **(A)** RT-qPCR analysis of *PIF4* transcript levels in Col-0, *bbx5-1* and *BBX5pro:BBX5-GFP #1* seedlings. Plants were grown in WL for 5 d, then transferred to low R/FR for 0, 1, 3, 6, 12, and 24 **h.** Values are means ± SD (n = 3). Asterisks indicate significant differences (*, *P* < 0.05; **, *P* < 0.01), as determined by two-tailed Student’s t-test. **(B)** Immunoblots analysis of BBX5-GFP protein levels in Col-0 and *bbx5-1* seedlings. Plants were grown in WL for 5 d, then transferred to low R/FR for 0, 1, 3, 6, 12, and 24 h. *pif4-2* was used as the negative control. Actin was used as the loading control. Numbers below the immunoblots indicate the relative intensities of BBX5-GFP bands normalized to those of loading controls, and the ratio was set to 1 for the first lane of each group. **(C)** Schematic representation of various constructs used in the transient transfection assays in *Nicotiana benthamiana* leaves. **(D)** Dual‐LUC assays showing that BBX5 activated the *PIF4pro:LUC* reporter. Values are means ± SD (n = 3). Asterisks indicate significant differences (**, *P* < 0.01), as determined by two-tailed Student’s t-test. **(E)** Schematic representation of the *PIF4* promoter with the location of the *G‐box* and *GT1* motif. Dark lines with numbers indicate regions examined by ChIP-qPCR. **(F)** ChIP-qPCR assays showing that BBX5 bindsto the *PIF4* promoter regions *in vivo*. Col-0 and *35Spro:BBX5-Flag* seedlings were grown in WL for 5 d, then either kept in WL or transferred to low R/FR for an additional 1 h respectively. Values are means ± SD (n = 3). Asterisks indicate significant differences (**, *P* < 0.01), as determined by two-tailed Student’s t-test. **(G-H)** Hypocotyl phenotypes and length of Col-0, *bbx5-1*, *pif4-2*, *bbx5-1 pif4-2*, *BBX5pro:BBX5-GFP #1* and *BBX5pro:BBX5-GFP pif4-2 #1* seedlings. Plants were grown in WL for 3 d, then either kept in WL or transferred to low R/FR conditions for an additional 3 d. Scale bar = 1 mm. Values are means ± SE (n ≥ 20). Letters above the bars indicate significant differences (*P* < 0.05), as determined by one-way ANOVA with Tukey’s post hoc analysis.

Low R/FR induces elevated levels of the growth-promoting factor PIF4 at both the transcriptional and protein levels [16,27]. We next examined whether BBX5 regulates the expression of *PIF4* under low R/FR conditions. The transcript levels of *PIF4* were decreased in *bbx5–1*, but increased in *BBX5pro:BBX5-GFP #1* when transferred to low R/FR for 1, 3 and 6 h (Fig 4A). Interestingly, the expression of *PIF4* in *bbx5–1* and *BBX5pro:BBX5-GFP #1* was comparable to that in Col-0 after 12 and 24 h of low R/FR irradiation (Fig 4A). Low R/FR triggers the accumulation of PIF4, consistent with previous studies [16]. The PIF4 protein levels were reduced in *bbx5–1* upon exposure to low R/FR for 1, 3 and 6 h, and became similar to those in Col-0 after 12 and 24 h of low R/FR treatment (Fig 4B). These results suggest that BBX5 positively regulates the *PIF4* transcription and hence contributes to the accumulation of PIF4 proteins specifically after short-term, but not prolonged, low R/FR exposure. Consistently, BBX5 activated the *PIF4pro:LUC* reporter when transiently co-expressed in *Nicotiana benthamiana* leaves (Fig 4C-D). We next performed ChIP-qPCR assays using Col-0 and *35Spro:BBX5-Flag* transgenic seedlings (S8 Fig). BBX5 proteins were enriched at the *PIF4*-*P2* promoter region under WL, whereas enrichment was observed at both *PIF4*-*P2* and -*P3* promoter regions under low R/FR conditions (Fig 4E-F). These results suggest that BBX5 associates with the promoter region of *PIF4* and activates its expression, thereby leading to the accumulation of PIF4 during early-stage shade exposure.

To explore the genetic relationship between *BBX5* and *PIF4*, we generated the *bbx5–1 pif4–2* double mutant and introduced the *pif4–2* mutation into the *BBX5pro:BBX5-GFP #1* line by genetic crossing. Both *bbx5–1* and *pif4–2* single mutants showed shortened hypocotyls, whereas the hypocotyl length of the *bbx5–1 pif4–2* double mutant seedlings was significantly shorter than that of *bbx5–1* and *pif4–2* grown in low R/FR (Fig 4G-H). The hypocotyls of *BBX5pro:BBX5-GFP pif4–2 #1* were slightly shorter than those of *BBX5pro:BBX5-GFP #1* grown in low R/FR (Fig 4G-H). These genetic results indicate that *BBX5* likely acts independently of *PIF4* in regulating SAS.

### BBX5 binds to the promoters of *YUC8* and *IAA19* and activates their transcription under low R/FR conditions

**Fig 5:**
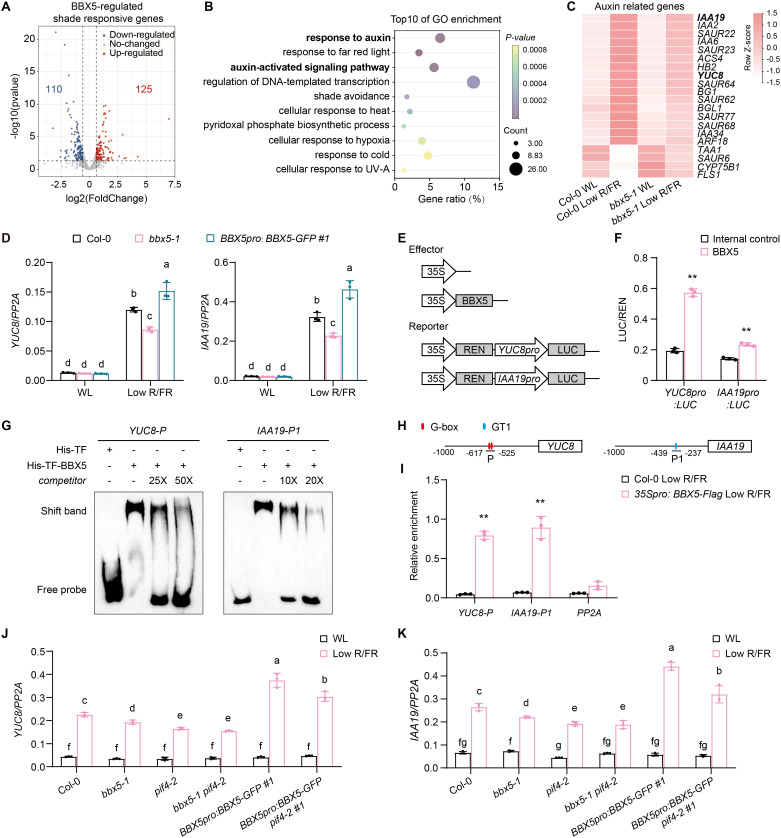
BBX5 associates with the promoters of *YUC8* and *IAA19* to activate their transcription in low R/FR. **(A**) Volcano plots showing the differentially expressed shade responsive genes in Col-0 and *bbx5-1* seedlings. Blue dots indicate significantly downregulated genes in *bbx5-1* vs Col-0 in low R/FR. Red dots indicate significantly upregulated genes in *bbx5-1* vs Col-0 in low R/FR. **(B)** Gene ontology (GO) analysis of 235 BBX5-regulated shade responsive genes. For each point, the size is proportional to the number of genes, and the colors represent the *P*-value. **(C)** Heatmaps showing the relative expression changes (*z*-normalized) of auxin-related genes regulated by BBX5. The values used to generate the heatmaps are the average TPMs of three independent RNA-seq replicates. **(D)** RT-qPCR analysis of *YUC8* and *IAA19* transcript levels in Col-0, *bbx5-1* and *BBX5pro:BBX5-GFP #1* seedlings. Plants were grown in WL for 5 d, then either kept in WL or transferred to low R/FR for an additional 1 h respectively. Values are means ± SD (n = 3). Letters above the bars indicate significant differences (*P* < 0.05), as determined by one-way ANOVA with Tukey’s post hoc analysis. **(E)** Schematic representation of various constructs used in the transient transfection assays in *Nicotiana benthamiana* leaves. **(F)** Dual‐LUC assay showing that BBX5 activated the *YUC8pro:LUC* and *IAA19pro:LUC* reporters. Values are means ± SD (n = 3). Asterisks indicate significant differences (**, *P* < 0.01), as determined by two-tailed Student’s t-test. **(G)** EMSA showing that BBX5 binds to *YUC8* and *IAA19* promoter subfragments *in vitro*. 6 × His-TF protein served as the negative control. The “+” and “−” indicate presence and absence, respectively. **(H)** Schematic representation of the *YUC8* and *IAA19* promoter with the location of the *G‐box* and *GT1* motif. Dark lines with numbers indicate regions examined by ChIP-qPCR. **(I)** ChIP-qPCR assays showing that BBX5 binds to the *YUC8* and *IAA19* promoter regions *in vivo*. Col-0 and *35Spro:BBX5-Flag* seedlings were grown in WL for 5 d, then transferred to low R/FR for 1 h. Values are means ± SD (n = 3). Asterisks indicate significant differences (**, *P* < 0.01), as determined by two-tailed Student’s t-test. **(J-K)** RT-qPCR analysis of *YUC8* and *IAA19* transcript levels in Col-0, *bbx5-1*, *pif4-2*, *bbx5-1 pif4-2*, *BBX5pro:BBX5-GFP #1* and *BBX5pro:BBX5-GFP pif4-2 #1* seedlings. Plants grown in WL for 5 d, then either kept in WL or transferred to low R/FR for an additional 1 h respectively. Values are means ± SD (n = 3). Letters above the bars indicate significant differences (*P* < 0.05), as determined by one-way ANOVA with Tukey’s post hoc analysis.

To analyze the BBX5-regulated genes, we performed RNA-seq experiments using Col-0 and *bbx5–1* grown in WL or exposed to low R/FR for 1 h. The transcription of approximately 2,255 genes (fold change > 1.5, *P* < 0.05) was significantly altered in Col-0 after 1 h low R/FR exposure compared with that in WL, and these genes are hereinafter referred to as shade-responsive genes (S1 Table). Of these shade-responsive genes, 110 were downregulated in *bbx5–1*, and 125 were upregulated in *bbx5–1* compared with Col-0 in low R/FR (Fig 5A and S2 Table). Gene Ontology (GO) enrichment analysis revealed that BBX5-regulated shade-response genes were involved in multiple biological responses including response to auxin, response to far-red light and auxin-activated signaling pathways (Fig 5B). Among these 235 BBX5-regulated shade-responsive genes, 20 are involved in auxin biosynthesis and signaling pathways including *YUC8* and *IAA19* (Fig 5C).

*YUC8* encodes a flavin-containing monooxygenase that catalyzes the biosynthesis of auxin [34], and IAA19 is a key regulator of auxin signaling [35]. Real-time qPCR analysis revealed that the expression of *YUC8* and *IAA19* was decreased in *bbx5–1*, but increased in *BBX5pro:BBX5-GFP* #1 in low R/FR (Fig 5D). Consistently, BBX5 activated the *YUC8pro:LUC* and *IAA19pro:LUC* reporters when transiently expressed in *Nicotiana benthamiana* leaves (Fig 5E-F). We next analyzed the promoter sequences of *YUC8* and *IAA19.* The *YUC8* promoter contains two typical *G-box* motifs within a 32-bp region (-603 to -562 bp), and the *IAA19* promoter region possesses one *GT1* and one *G-box* motif (S9 Fig). We performed an *in vitro* Electrophoretic Mobility Shift Assay (EMSA) to test whether BBX5 directly binds to these DNA sites. Purified recombinant His-Trigger Factor (TF)-BBX5 bound to the biotin-labeled *YUC8pro* (-628 to -538 bp) containing two *G-box* motifs and the *IAA19pro-P1* (-358 to -237 bp) containing one *GT1* motif, but did not bind to the *IAA19pro-P2* DNA sub-fragments (-191 to -144 bp) containing one *G-box* (Figs 5G and S10). The negative control His-TF showed no binding to these probes. As the amounts of non-biotin-labeled *YUC8pro* or *IAA19pro-P1* DNA fragments (competitor) increased in the reactions, the binding of His-TF-BBX5 to these biotin-labeled probes clearly decreased (Fig 5G). Next, we performed ChIP-qPCR assays using Col-0 and *35Spro:BBX5-Flag* transgenic plants grown in low R/FR*.* BBX5-Flag proteins were significantly enriched at the promoter regions of *YUC8* (harboring two *G-box* motifs) and *IAA19* (harboring one *GT1* motif) (Fig 5H-I). Together, these results suggest that BBX5 directly binds to the promoter regions of *YUC8* and *IAA19* to activate their transcription in response to low R/FR.

Previous studies have demonstrated that PIF4 directly activates the expression of *YUC8* and *IAA19* [36,37]*.* We thus carried out real-time qPCR assays to examine whether PIF4 and BBX5 co-regulate the transcription of these two genes in low R/FR. The transcript levels of *YUC8* and *IAA19* in *bbx5–1 pif4–2* were comparable to those in *pif4–2,* in which the expression of these two genes was slightly lower than those in Col-0 and *bbx5–1* under low R/FR. The expression of *YUC8* and *IAA19* was significantly increased in *BBX5pro:BBX5-GFP* #1 and *BBX5pro:BBX5-GFP pif4–2* #1. However, their transcript levels were lower in *BBX5pro:BBX5-GFP pif4–2* #1 than in *BBX5pro:BBX5-GFP* #1 under low R/FR (Fig 5J-K). These results suggest that BBX5 and PIF4 may coregulate the expression of *YUC8* and *IAA19* under low R/FR conditions.

## Discussion

Shade-intolerant plants undergo SAS when grown in crowded environments [4,5]. The photoreceptor phyB serves as the primary sensor of low R/FR light signals. Low R/FR triggers the conversion of phyB from its active Pfr form to inactive Pr form, thus releasing the suppression of PIF4 [12,13,38]. In addition, low R/FR induces the transcription of *PIF4*, thus partially contributing to the accumulation of PIF4 [27]. Accumulated PIF4 upregulates the expression of auxin biosynthetic and signaling genes to promote plant growth [19,36,37,39,40]. In this study, we revealed that BBX5 transcriptionally upregulated *PIF4* during the early phase of low R/FR exposure, and directly activated the expression of the auxin biosynthetic gene *YUC8* and the auxin signaling gene *IAA19.*

phyB interacted with BBX5 in WL (Fig 2B). The majority of phyB remains in the active Pfr form that localizes in the nucleus in WL, while low R/FR triggers the photoconversion of Pfr to Pr, leading to its translocation to the cytoplasm [13,38]. Thus, BBX5 likely preferentially interacts with phyB Pfr in the nucleus. Low R/FR induces conformational switching of phyB and its subsequent nucleocytoplasmic partitioning, thereby presumably dissociating the phyB-BBX5 complex. Loss of phyB function led to decreased BBX5 accumulation in WL, R and low R/FR (Figs 2C and S3), indicating that phyB is necessary for BBX5 stabilization irrespective of light conditions. Notably, although phyB was scarcely detectable in BBX5 immunoprecipitants from *Arabidopsis* seedlings exposed to low R/FR (Fig 2B), a small residual pool of phyB Pfr might still associate with BBX5, thereby maintaining its stability.

The E3 ligase COP1 undergoes nuclear translocation from the cytoplasm in response to low R/FR [41]. Accumulated nuclear COP1 targets LONG HYPOCOTYL IN FAR-RED (HFR1) for ubiquitination and subsequent degradation, consequently increasing the activity of PIFs to promote growth [42,43]. COP1 also associates with BBX5 in plant cells. COP1-mediated degradation of BBX5 is enhanced by darkness and low R/FR signals (Figs 2E and S6). These results were consistent with the observations that the BBX5 protein levels were markedly reduced in darkness or during the initial period of low R/FR exposure compared to those in WL (S2B and S2D Fig). BBX5 regulated the expression of a large number of genes and promoted SAS in response to shade signals (Figs 1 and 5A-C), establishing its role as a positive regulator of SAS. COP1-mediated degradation of BBX5 under early shade conditions may serve to prevent exaggerated SAS in plants.

phyB acts upstream of COP1 and inactivates it by disrupting COP1-SPAs complex formation [44]. The BBX5-GFP protein level was markedly reduced in *BBX5pro:BBX5-GFP phyB-9* #1 compared to *BBX5pro:BBX5-GFP* #1 (Fig 2C), likely due to loss of phyB function and the consequent increase in COP1 activity. phyB and COP1 acted antagonistically to control BBX5 stability. phyB positively controlled the accumulation of BBX5 (Fig 2), while COP1 promoted its degradation via the 26S proteasome system (Fig 3). BBX5 levels were markedly reduced but remained detectable in *Arabidopsis* seedlings during early low R/FR exposure, and subsequently reaccumulated under prolonged shade conditions (S2D Fig). These dynamics suggest that BBX5 abundance is regulated by multiple factors, including phyB, COP1 and likely additional as-yet-unidentified factor(s) under shade conditions.

BBX5 associated with the *PIF4* promoter and upregulated its expression, hence leading to the accumulation of PIF4 during early phase of low R/FR irradiation (Fig 4). BBX7 and BBX8 also upregulate the expression of *PIF4* through CCA1 and LHY in shade [27]. The precise molecular mechanisms by which these three closely related family members coordinately and synergistically regulate *PIF4* transcription await further investigation. PIF4 directly activates the transcription of numerous auxin biosynthetic and signaling genes, leading to elevated auxin levels and auxin-mediated growth [19,36,37]. Transcriptome analysis revealed that BBX5 positively controlled the expression of 20 genes involved in auxin biosynthetic and signaling genes in shade (Fig 5C). The transcriptional activation of these genes by BBX5 is at least partially attributable to elevated PIF4 protein levels. In addition to activating *PIF4* transcription, BBX5 also directly bound to the promoter regions of the auxin biosynthetic gene *YUC8* and the signaling gene *IAA19,* and activated their transcription (Fig 5D-I). Consistently, the *bbx5–1 pif4–2* double mutant exhibited reduced sensitivity to shade relative to the *bbx5–1* and *pif4–2* single mutants (Fig 4G-H). Thus, BBX5 employs at least two distinct molecular mechanisms to promote auxin-mediated growth in *Arabidopsis*.

**Fig 6:**
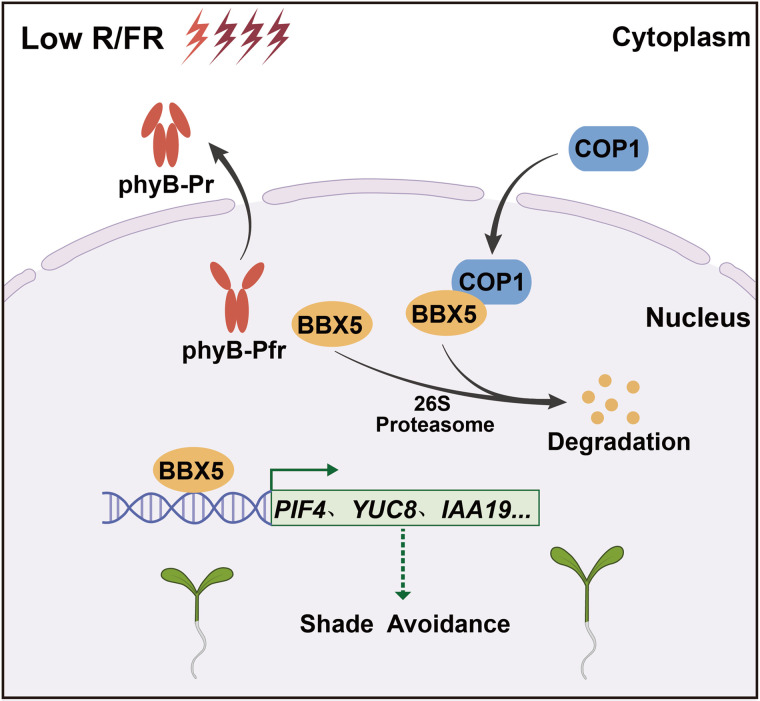
A proposed working model showing how BBX5 promotes shade avoidance in *Arabidopsis.* Upon shade irradiation, the remaining phyB stabilizes BBX5, whereas the E3 ubiquitin ligase COP1 promotes the degradation of BBX5 via the 26S proteasome system in the nucleus. BBX5 directly targets *PIF4*, *YUC8* and *IAA19* to activate their transcription, thereby promoting shade avoidance in *Arabidopsis*.

Collectively, our study revealed that the photoreceptor phyB stabilizes BBX5, whereas the E3 ubiquitin ligase COP1 de-stabilizes it. BBX5 directly targets *PIF4*, *YUC8* and *IAA19* to activate their expression, thereby promoting SAS in response to shade (Fig 6). Our results highlight a molecular framework for shade-initiated BBX5-*PIF4*-*YUC8*/*IAA19* signaling cascade that promotes plant growth and development.

## Materials and methods

### Plant materials and growth conditions

The *cop1–6* [45], *phyB‐9* [46], *phyB-CFP* [47], *pif4‐2* [48], *bbx5–1* and *bbx5–2* mutants, *BBX5pro:BBX5-GFP #1*, *BBX5pro:BBX5-GFP #2* and *35Spro:BBX5-Flag* (this study) transgenic lines are in the *Arabidopsis* Columbia‐0 (Col‐0) ecotype. Double‐mutant/transgenic plants were generated by genetic crossing, and homozygous lines were verified by PCR genotyping or antibiotic screen. Seeds were surface‐sterilized with 20% NaClO and sown on 0.5 × Murashige and Skoog (½MS) medium containing 1% sucrose and 0.8% agar. After stratification in darkness at 4°C for 3 d, the plates were then either kept in WL [38 μmol·m ⁻ ²·s ⁻ ^1^, R (15.73 μmol·m ⁻ ²·s ⁻ ¹)/FR (1.97 μmol·m ⁻ ²·s ⁻ ¹) = 7.98] or transferred to low R/FR [R (19.23 μmol·m ⁻ ²·s ⁻ ¹)/FR (81.01 μmol·m ⁻ ²·s ⁻ ¹) = 0.24] in a growth chamber (HiPoint, China) maintained at 22°C. The hypocotyl length was measured using IMAGE J software.

### Generation of *bbx5* mutant and *BBX5*-overexpressing transgenic plants

Generation of *bbx5* mutant using CRISPR/Cas9 technique was performed as previously described [49]. 23-bp target sites (5’-N20NGG-3’) within exons of genomic DNA sequences of *BBX5* were manually searched and identified. Each of these sites was evaluated for target specificities on the website of potential off-target finder (http://www.rgenome.net/cas-offinder/). Two independent sgRNA target sites of *BBX5* were sub-cloned into *pHEE401E* vector. These constructs were transformed into *Agrobacterium* strain *GV3101*, and then introduced into Col-0 plants via the floral dip method. T_1_ transgenic plants were selected on ½MS containing 25 mg/L hygromycin. The *BBX5* promoter and coding sequence were ligated into the *pCAMBIA1300* vector to produce *pCAMBIA1300-BBX5pro:BBX5-GFP* construct. The *BBX5* coding sequence was ligated into the *pCAMBIA1307* vector to produce *pCAMBIA1307–35Spro:BBX5-Flag* construct. Those constructs were transformed into *Agrobacterium* strain *GV3101* respectively, and then introduced into Col-0 plants via the floral dip method. Transgenic plants were screened on ½MS containing 25 mg/L hygromycin. Primers used for plasmid constructions are listed in S3 Table.

### Firefly luciferase complementation imaging assays

The firefly luciferase complementation imaging (LCI) transient expression assay was performed as previously described [50]. The coding sequences of *MBP* and *BBX5* were ligated into the *pCAMBIA1300‐LUC*^*N*^ vector to produce *pCAMBIA1300-MBP-LUC*^*N*^ and *pCAMBIA1300-BBX5-LUC*^*N*^ constructs. The coding sequences of *GST*, *phyB* and *COP1* were ligated into the *pCAMBIA1300-LUC*^*C*^ vector to produce *pCAMBIA1300-LUC*^*C*^*-GST*, *pCAMBIA1300-LUC*^*C*^*-phyB* and *pCAMBIA1300-LUC*^*C*^*-COP1* constructs. Those constructs were transformed into *Agrobacterium* strain *GV3101* respectively, and then the *Agrobacterium* strains *GV3101* harboring different constructs were infiltrated into tobacco leaves respectively. The *pCAMBIA1300-MBP‐LUC*^*N*^ and *pCAMBIA1300‐LUC*^*C*^*-GST* constructs were used as negative controls. After a 24 h incubation in darkness at 22°C and an additional 24–36 h incubation under a 16 h light/8 h dark photoperiod, the 0.33 mmol/L D‐luciferin (40901ES, Yesaen, China) solution was sprayed onto the tobacco leaves. LUC activity was measured with the LB 985 NightSHADE Spectrum imaging system (Berthold, Germany). Primers used for plasmid constructions are listed in S3 Table.

### Co‐immunoprecipitation assays (Co-IP)

*BBX5pro:BBX5-GFP #1* seedlings were grown in darkness for 4 d, then one of the duplicates was treated with white light for 1 h, and the other was treated with low R/FR for 1 h. The total proteins were extracted with Co-IP buffer (150 mM NaCl, 10 mM MgCl_2_, 50 mM Tris-HCl pH 7.5, 1 mM EDTA, 0.2% (v/v) NP-40, 1 mM phenylmethylsulfonyl fluoride, 80 μM MG132, 1 × EDTA-free protease inhibitor cocktail, 1 × EDTA-free phosphatase inhibitor cocktail). After centrifugation, the supernatant was treated with WL or low R/FR for an additional 5 min and then incubated with anti-GFP Magarose Beads (SM038005, Smart-Lifesciences, China) for 3 h. The beads were then gently washed five times with Co-IP buffer at 4°C. The Input and IP-proteins were analyzed by western blotting with anti-GFP (M20004, Abmart, China), anti-phyB [51] and anti-COP1 [52]. Primers used for plasmid constructions are listed in S3 Table.

### Yeast two-hybrid assay

Yeast two-hybrid assays were performed using the Matchmaker GAL4 Two-Hybrid Systems as described in the Yeast Protocols Handbook (BD Clontech). The full length, truncated and mutated coding sequences of *BBX5* were ligated into the *pGADT7* vector to produce *pGADT7-BBX5, pGADT7-BBX5-N, pGADT7-BBX5-M, pGADT7-BBX5-C* and *pGADT7-BBX5*^*VP/AA*^ constructs. The coding sequence of COP1 was ligated into the *pGBKT7* vector to produce *pGBKT7-COP1* construct. The indicated combinations of *pGADT7* and *pGBKT7*-fused constructs were co-transformed into yeast strain *AH109*. The empty *pGADT7* and *pGBKT7* vectors were used as negative controls. Transformants were selected and grown on stringent selective synthetic defined (SD) media lacking Trp, Leu and His for the test of protein interactions. Yeast cells were plated on nonselective SD media lacking Trp and Leu to show the successful transformation of the constructs. Primers used for plasmid constructions are listed in S3 Table.

### CHX and MG132 treatment

For cycloheximide (CHX) treatment, *BBX5pro:BBX5-GFP* #1, *BBX5pro:BBX5-GFP phyB-9* #1 and *BBX5pro:BBX5-GFP cop1–6* #1 seedlings were grown in white light for 5 d, then submerged in 1/2 MS liquid medium containing 500μM CHX (01810, Sigma-Aldrich, USA) under WL or low R/FR conditions. Seedlings were collected at indicated time points. For MG132 treatment, *BBX5pro:BBX5-GFP* #1 seedlings were grown in white light for 5 d, then submerged in 1/2 MS liquid medium containing 50, 100, or 200 μM MG132 (S2691, Selleck, China) for 3 h. Seedlings were collected after 1 h of low R/FR treatment. Total protein was subsequently extracted and subjected to immunoblot analysis.

### Dual-luciferase reporter system

The *BBX5* coding sequence was ligated into the *pCAMBIA1307* vector to produce *pCAMBIA1307–35Spro:BBX5-Flag* construct. The promoters of *PIF4*, *YUC8* and *IAA19* were ligated into the *pGreen0800II-LUC* vector to produce *pGreen0800II-PIF4pro:LUC*, *pGreen0800II-YUC8pro:LUC* and *pGreen0800II-IAA19pro:LUC* constructs. Those constructs were transformed into *Agrobacterium* strain *GV3101* respectively, and then the *Agrobacterium* strains *GV3101* harboring different constructs were infiltrated into tobacco leaves. The empty *pCAMBIA1307* vector was used as a negative control. Firefly LUC and Renilla LUC (Ren) were detected using the Dual-LUC Reporter Assay System (DL101, Vazyme, China) according to the manufacturer’s instructions. Primers used for plasmid constructions are listed in S3 Table.

### Electrophoretic Mobility Shift Assay

The *BBX5* coding sequence was ligated into the *pCold-His-TF* vector to produce *pCold-His-TF-BBX5* construct, and then transformed and expressed in *E.coli BL21* cells. The purified recombinant His-TF-BBX5 proteins were used for the EMSA. The purified His-TF proteins were used as a negative control. Biotin-labeled probes were obtained with the EMSA Probe Biotin Labeling Kit (GS008, Beyotime, China). The binding reaction was performed using Chemiluminescent EMSA Kit (GS009; Beyotime, China) according to the manufacturer’s protocol. The specific probes used for EMSA are listed in S3 Table.

### RNA-sequencing

For RNA-seq analysis, Col-0 and *bbx5–1* seedlings were grown in WL for 5 d, then one of the duplicates was treated with white light for 1 h, and the other was treated with low R/FR for 1 h. Three independent biological replicates were performed for each line and condition. Seedlings were collected and total RNA was extracted from snap-frozen tissues using VeZol-Pure Total RNA Isolation Kit (RC202, Vazyme, China) according to the manufacturer’s instructions. Total RNA was processed following standard protocols of the Novogene Co. for preparing Illumina RNA sequencing (RNA-Seq) libraries and then sequenced on a NovaSeq 6000 platform to generate paired-end 150 bp reads. RNA-seq reads were cleaned and aligned to the *Arabidopsis* genome (TAIR10) using HISAT2 with default parameters. Reads per gene were counted with HTSeq. Differential expression analysis was performed using DESeq2 with foldchange>1.5 and *P*-value < 0.05. The shade responsive genes in Col-0 (Col-0 low R/FR vs. Col-0 WL) are listed in S1 Table. Shade responsive genes regulated by BBX5 (*bbx5–1* low R/FR vs. Col-0 low R/FR) are listed in S2 Table. Gene ontology (GO) analysis was performed with DAVID (https://david.ncifcrf.gov/).

### Chromatin immunoprecipitation-qPCR assay

The chromatin immunoprecipitation (ChIP) assays were performed as described previously [27]. Col-0 and *35Spro:BBX5-Flag* seedlings were grown in white light for 5 d, then either kept in WL or transferred to low R/FR for an additional 1 h. Three independent biological replicates were performed for each line. Seedlings were fixed with 1% formaldehyde and ground into a fine powder in liquid nitrogen. The nuclei in the powder were extracted through Extraction buffer I (0.4 M sucrose, 10 mM Tris-HCl, pH 8.0, 10 mM MgCl_2_, 5 mM β-mercaptoethanol, 0.1 mM PMSF, and 1 × EDTA-free protease inhibitor cocktail), Extraction buffer II (0.25 M sucrose, 10 mM Tris-HCl pH 8.0, 10 mM MgCl_2_, 1% (v/v) Triton X-100, 5 mM β-mercaptoethanol, 0.1 mM PMSF, and 1 × EDTA-free protease inhibitor cocktail) and Extraction buffer III (1.7 M sucrose, 10 mM Tris-HCl pH 8.0, 2 mM MgCl_2_, 0.15% (v/v) Triton X-100, 5 mM β-mercaptoethanol, 0.1 mM PMSF, and 1 × EDTA-free protease inhibitor cocktail). The collected nuclei were resuspended in Nuclei lysis buffer (50 mM Tris-HCl pH 8.0, 10 mM EDTA, 1% (w/v) SDS, and 1 × EDTA-free protease inhibitor cocktail) and then sonicated until the average chromatin size was approximately 300 bp. The sonicated chromatin was diluted 10-fold with ChIP dilution buffer (1.1% (v/v) Triton X-100, 1.2 mM EDTA, 16.7 mM Tris-HCl pH 8.0, 167 mM NaCl) and then incubated overnight at 4°C with anti-Flag antibody (M185, MBL, Japan). Approximately 2% of diluted chromatin was reverse cross-linked and served as input DNA control. The chromatin-antibody mixture was incubated with Protein A/G (#88802, Thermo Scientific, USA). The beads were then washed, and the immunoprecipitates were eluted from the beads and reverse cross-linked. Purified immunoprecipitated DNA and input DNA were used for RT-qPCR. Primers used in this experiment are listed in S3 Table.

### Statistical analysis

Statistical analyses were performed in Microsoft Excel, GraphPad Prism version 5.0 or through an online website (http://astatsa.com/OneWay_Anova_with_TukeyHSD/). For comparing two groups, statistical analyses were carried out using a two-tailed student’s t-test (*, *P* < 0.05; **, *P* < 0.01; ***, *P* < 0.001). For comparing multiple groups, statistical analyses were carried out using one-way ANOVA with Tukey’s post-hoc analysis (*P* < 0.05).

## Supporting information

S1 Fig Mutations in *bbx5* alleles and BBX5-GFP protein levels in *BBX5-GFP* transgenic seedlings. **(A)** The DNA sequence alignment shows altered bases in *bbx5–1* and *bbx5–2* mutants. Nucleic acid mutations were indicated in red. **(B)** Protein structures of BBX5, BBX5–1 and BBX5–2. The numbers indicate the positions of amino acids. **(C)** Immunoblots showing the BBX5-GFP protein levels in *BBX5pro:BBX5-GFP #1* and *BBX5pro:BBX5-GFP #2* transgenic seedlings. Plants were grown in the continuous WL for 5 d. Col-0 was used as the negative control. Actin was used as the loading control.
(TIF)

S2 Fig The expression pattern of BBX5 in response to WL and low R/FR at the transcriptional and protein levels. **(A-B)** RT-qPCR analysis of *BBX5* transcript levels in Col-0 seedlings in response to WL and low R/FR. Plants were grown in dark for 4 d and then transferred to WL for 0, 1, 3, 6, 12, and 24 h (**A**), or grown in WL for 5 d and then transferred to low R/FR for 0, 1, 3, 6, 12, and 24 h (**B**). Values are means ± SD (n = 3). Letters above the bars indicate significant differences (*P* < 0.05), as determined by one-way ANOVA with Tukey’s post hoc analysis. **(C-D)** Immunoblots analysis of BBX5-GFP protein levels in *BBX5pro:BBX5-GFP #1* seedlings in response to WL and low R/FR. Plants were grown in dark for 4 d and then transferred to WL for 0, 1, 3, 6, 12, and 24 h (**C**), or grown in WL for 5 d and then transferred to low R/FR for 0, 1, 3, 6, 12, and 24 h (**D**). Col-0 was used as the negative control. Actin was used as the loading control. Numbers below the immunoblots indicate the relative intensities of BBX5-GFP bands normalized to those of loading controls, and the ratio was set to 1 for the first lane of each group.
(TIF)

S3 Fig phyB stabilizes BBX5 proteins in red light. Immunoblots showing the BBX5-GFP protein levels in *BBX5pro:BBX5-GFP #1* and *BBX5pro:BBX5-GFP phyB-9 #1* seedlings grown in red light. Plants were grown in red light for 4 d. Col-0 was used as the negative control. Actin was used as the loading control.
(TIF)

S4 Fig phyB has little effect on the transcription of *BBX5.* **(A)** RT-qPCR analysis of *BBX5* transcript levels in Col-0, *phyB-9* and *PBC* seedlings. Plants were grown in red light for 4 d, or grown in WL for 5 d and then either kept in WL or transferred to low R/FR for an additional 1 h respectively. **(B)** RT-qPCR analysis of BBX5 transcript levels in Col-0, *BBX5pro:BBX5-GFP #1* and *BBX5pro:BBX5-GFP phyB-9 #1* seedlings. Plants were grown in red light for 4 d, or grown in WL for 5 d and then either kept in WL or transferred to low R/FR for an additional 1 h respectively. Values are means ± SD (n = 3). Letters above the bars indicate significant differences (*P* < 0.05), as determined by one-way ANOVA with Tukey’s post hoc analysis.
(TIF)

S5 Fig phyB stabilizes BBX5 proteins by inhibiting the 26S proteasome-mediated degradation. Immunoblots analysis showing BBX5-GFP protein levels in *BBX5pro: BBX5-GFP #1* and *BBX5pro: BBX5-GFP phyB-9 #1* seedlings after GM132 and CHX treatment. Plants were grown in WL for 5 d, then pretreated with MG132 (200 μM) for 3 h, and subsequently incubated with CHX (500 μM) for 2 h under WL or low R/FR conditions respectively. Actin was used as the loading control. Numbers below the immunoblots indicate the relative intensities of BBX5-GFP bands normalized to those of loading controls, and the ratio was set to 1 for the first lane of each seedling.
(TIF)

S6 Fig COP1 promotes the degradation of BBX5 protein in the dark. Immunoblots showing the BBX5-GFP protein levels in *BBX5pro:BBX5-GFP #1* and *BBX5pro:BBX5-GFP cop1–6 #1* seedlings. Plants were grown in the dark for 4 d. Col-0 was used as the negative control. Actin was used as the loading control.
(TIF)

S7 Fig COP1 does not regulate the transcription of *BBX5.* RT-qPCR analysis of BBX5 transcript levels in Col-0, *BBX5pro:BBX5-GFP #1* and *BBX5pro:BBX5-GFP cop1–6 #1* seedlings. Plants were grown in dark for 4 d, or grown in WL for 5 d and then either kept in WL or transferred to low R/FR for an additional 1 h respectively. Values are means ± SD (n = 3). Letters above the bars indicate significant differences (*P* < 0.05), as determined by one-way ANOVA with Tukey’s post hoc analysis.
(TIF)

S8 Fig Overexpression of *BBX5-Flag* results in elongated hypocotyls in low R/FR. **(A)** Immunoblots showing the BBX5-Flag protein levels in *35Spro:BBX5-Flag* transgenic seedlings. Plants were grown in continuous WL for 5 d. Col-0 was used as the negative control. Actin was used as the loading control. **(B-C)** Hypocotyl phenotypes (**B**) and length (**C**) of Col-0, *bbx5–1* and *35Spro:BBX5-Flag* seedlings. Plants were grown in WL for 3 d, then either kept in WL or transferred to low R/FR conditions for 3 d. Values are means ± SE (n ≥ 20). Scale bar = 1 mm. Letters above the bars indicate significant differences (*P* < 0.05), as determined by one-way ANOVA with Tukey’s post hoc analysis.
(TIF)

S9 Fig Schematic representation of the *YUC8* and *IAA19* promoter. Schematic representation of the *YUC8* and *IAA19* promoter with the location of the *G‐box* and *GT1-motif*. The numbers indicate the positions of the *G-box* and *GT1* motifs.
(TIF)

S10 Fig BBX5 does not bind to the *IAA19pro-P2 in vitro.* EMSA showing that BBX5 did not bind to the *IAA19pro-P2* subfragments containing one *G-box* motif *in vitro*. The “+” and “−” indicate presence and absence, respectively.
(TIF)

S1 Table List of 2255 shade responsive genes. (XLSX)

S2 Table List of 235 shade responsive genes regulated by BBX5. (XLSX)

S3 Table List of primers used in this study. (XLSX)

S1 Data Underlying numerical data. (XLSX)
