# Supplementary figures and images for: Promotion of shade avoidance by BBX5 involves activation of PIF4 along with auxin biosynthetic and signaling genes
Source: PLoS Genet. 2026 Jun 5;22(6):e1012177. doi: 10.1371/journal.pgen.1012177 (PMC13252834; doi:10.1371/journal.pgen.1012177)

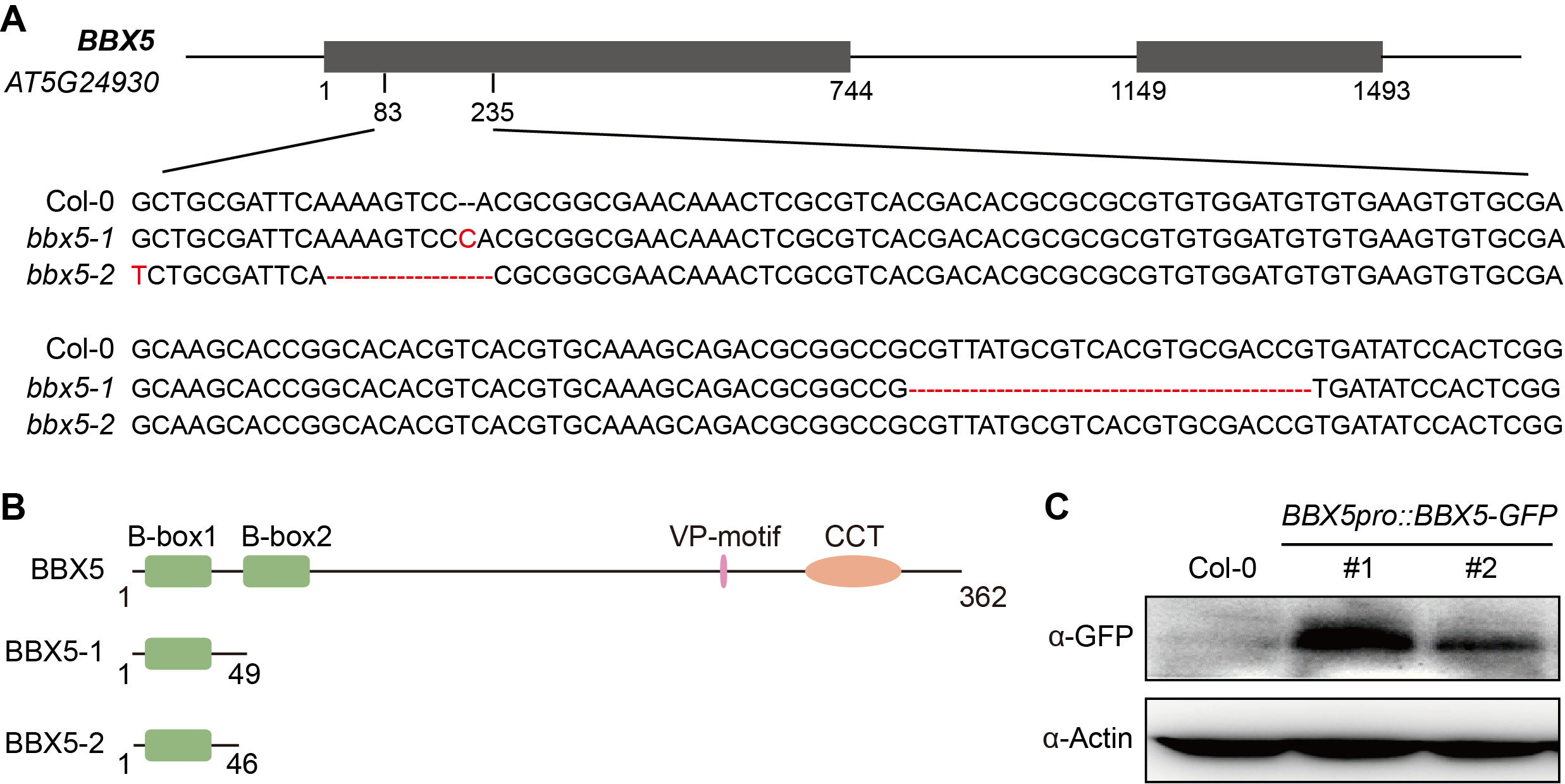

Supplement: S1 Fig — (A) The DNA sequence alignment shows altered bases in bbx5–1 and bbx5–2 mutants. Nucleic acid mutations were indicated in red. (B) Protein structures of BBX5, BBX5–1 and BBX5–2. The numbers indicate the positions of amino acids. (C) Immunoblots showing the BBX5-GFP protein levels in BBX5pro:BBX5-GFP #1 and BBX5pro:BBX5-GFP #2 transgenic seedlings. Plants were grown in the continuous WL for 5 d. Col-0 was used as the negative control. Actin was used as the loading control. (TIF) [file pgen.1012177.s001.tif]

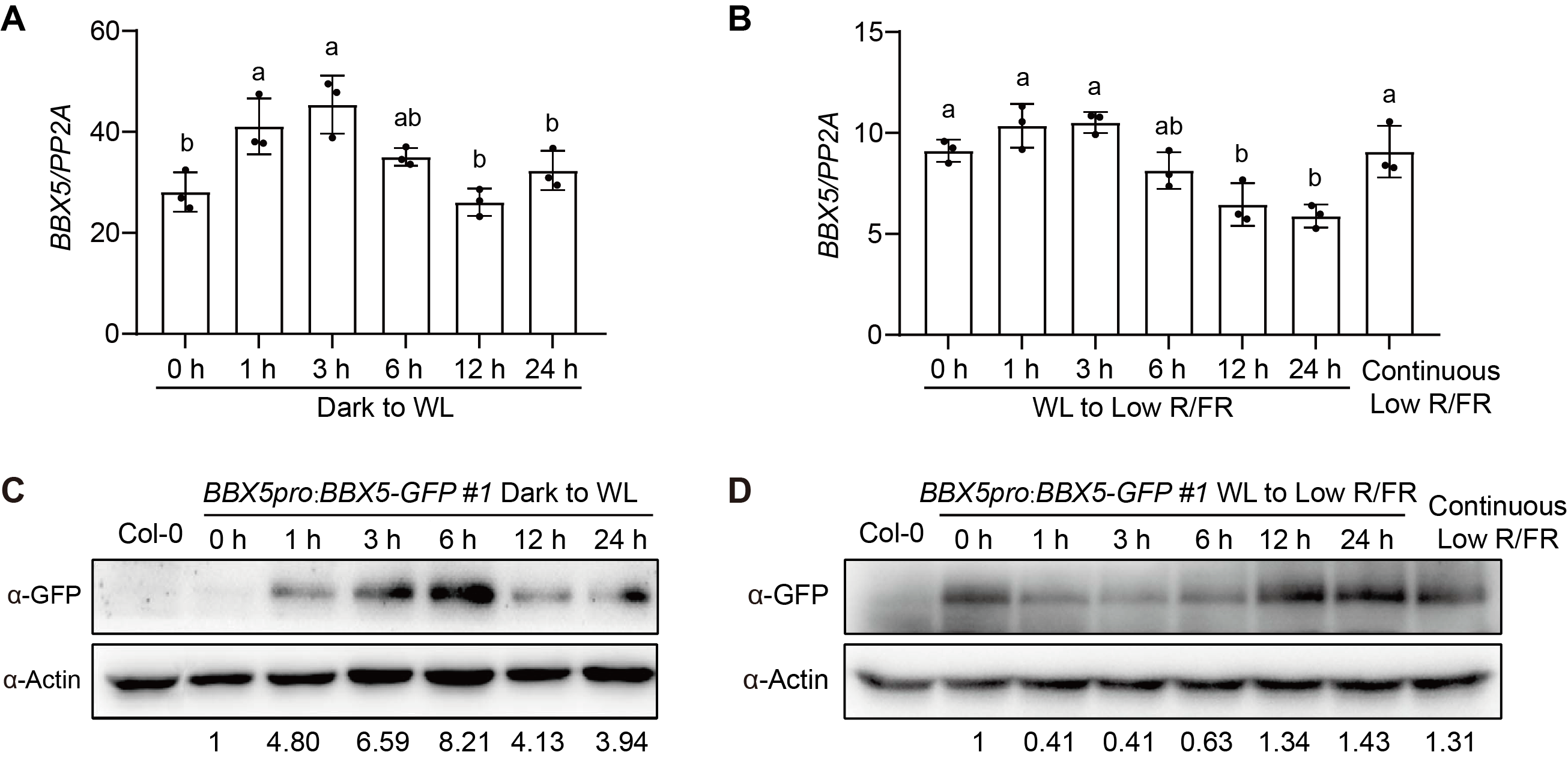

Supplement: S2 Fig — (A-B) RT-qPCR analysis of BBX5 transcript levels in Col-0 seedlings in response to WL and low R/FR. Plants were grown in dark for 4 d and then transferred to WL for 0, 1, 3, 6, 12, and 24 h (A), or grown in WL for 5 d and then transferred to low R/FR for 0, 1, 3, 6, 12, and 24 h (B). Values are means ± SD (n = 3). Letters above the bars indicate significant differences (P < 0.05), as determined by one-way ANOVA with Tukey’s post hoc analysis. (C-D) Immunoblots analysis of BBX5-GFP protein levels in BBX5pro:BBX5-GFP #1 seedlings in response to WL and low R/FR. Plants were grown in dark for 4 d and then transferred to WL for 0, 1, 3, 6, 12, and 24 h (C), or grown in WL for 5 d and then transferred to low R/FR for 0, 1, 3, 6, 12, and 24 h (D). Col-0 was used as the negative control. Actin was used as the loading control. Numbers below the immunoblots indicate the relative intensities of BBX5-GFP bands normalized to those of loading controls, and the ratio was set to 1 for the first lane of each group. (TIF) [file pgen.1012177.s002.tif]

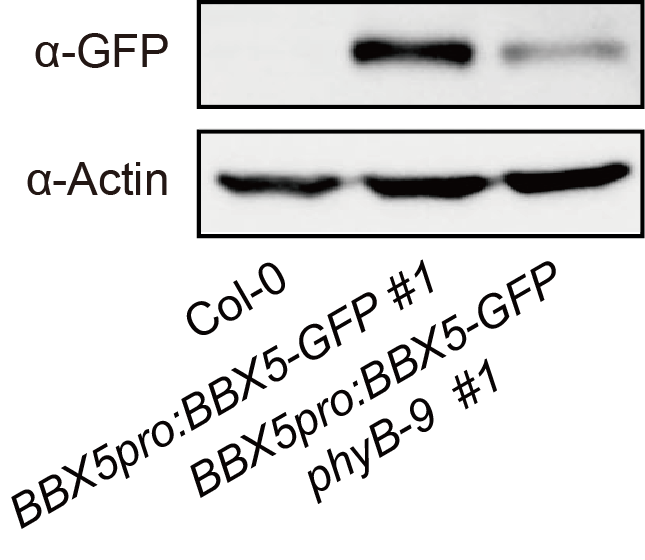

Supplement: S3 Fig — Immunoblots showing the BBX5-GFP protein levels in BBX5pro:BBX5-GFP #1 and BBX5pro:BBX5-GFP phyB-9 #1 seedlings grown in red light. Plants were grown in red light for 4 d. Col-0 was used as the negative control. Actin was used as the loading control. (TIF) [file pgen.1012177.s003.tif]

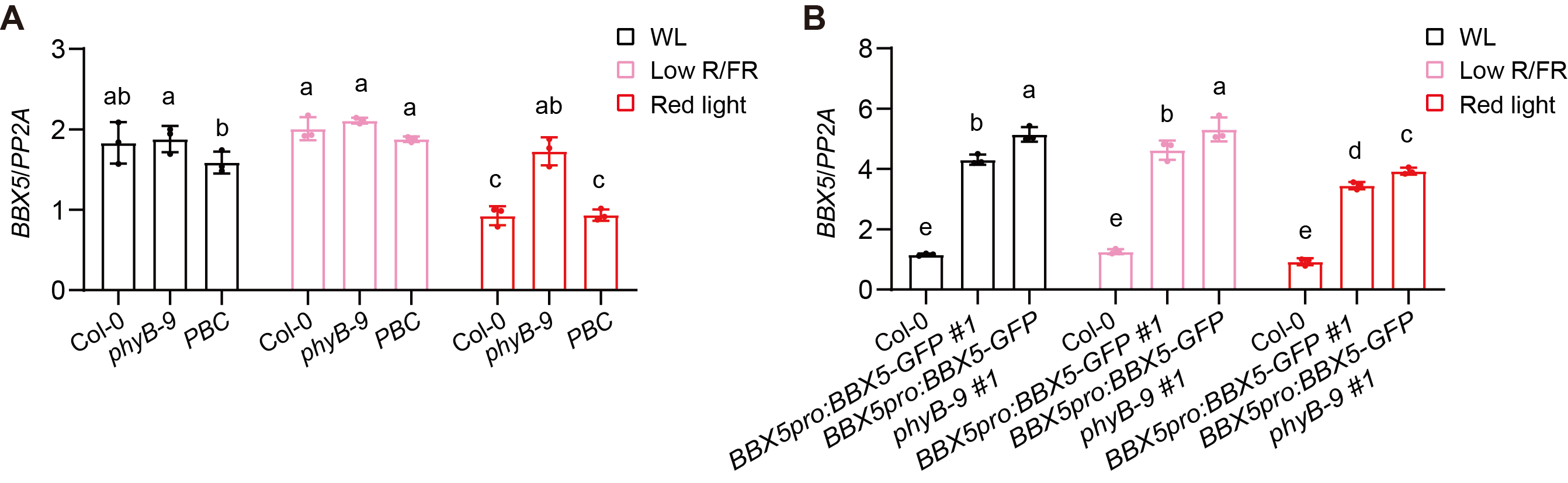

Supplement: S4 Fig — (A) RT-qPCR analysis of BBX5 transcript levels in Col-0, phyB-9 and PBC seedlings. Plants were grown in red light for 4 d, or grown in WL for 5 d and then either kept in WL or transferred to low R/FR for an additional 1 h respectively. (B) RT-qPCR analysis of BBX5 transcript levels in Col-0, BBX5pro:BBX5-GFP #1 and BBX5pro:BBX5-GFP phyB-9 #1 seedlings. Plants were grown in red light for 4 d, or grown in WL for 5 d and then either kept in WL or transferred to low R/FR for an additional 1 h respectively. Values are means ± SD (n = 3). Letters above the bars indicate significant differences (P < 0.05), as determined by one-way ANOVA with Tukey’s post hoc analysis. (TIF) [file pgen.1012177.s004.tif]

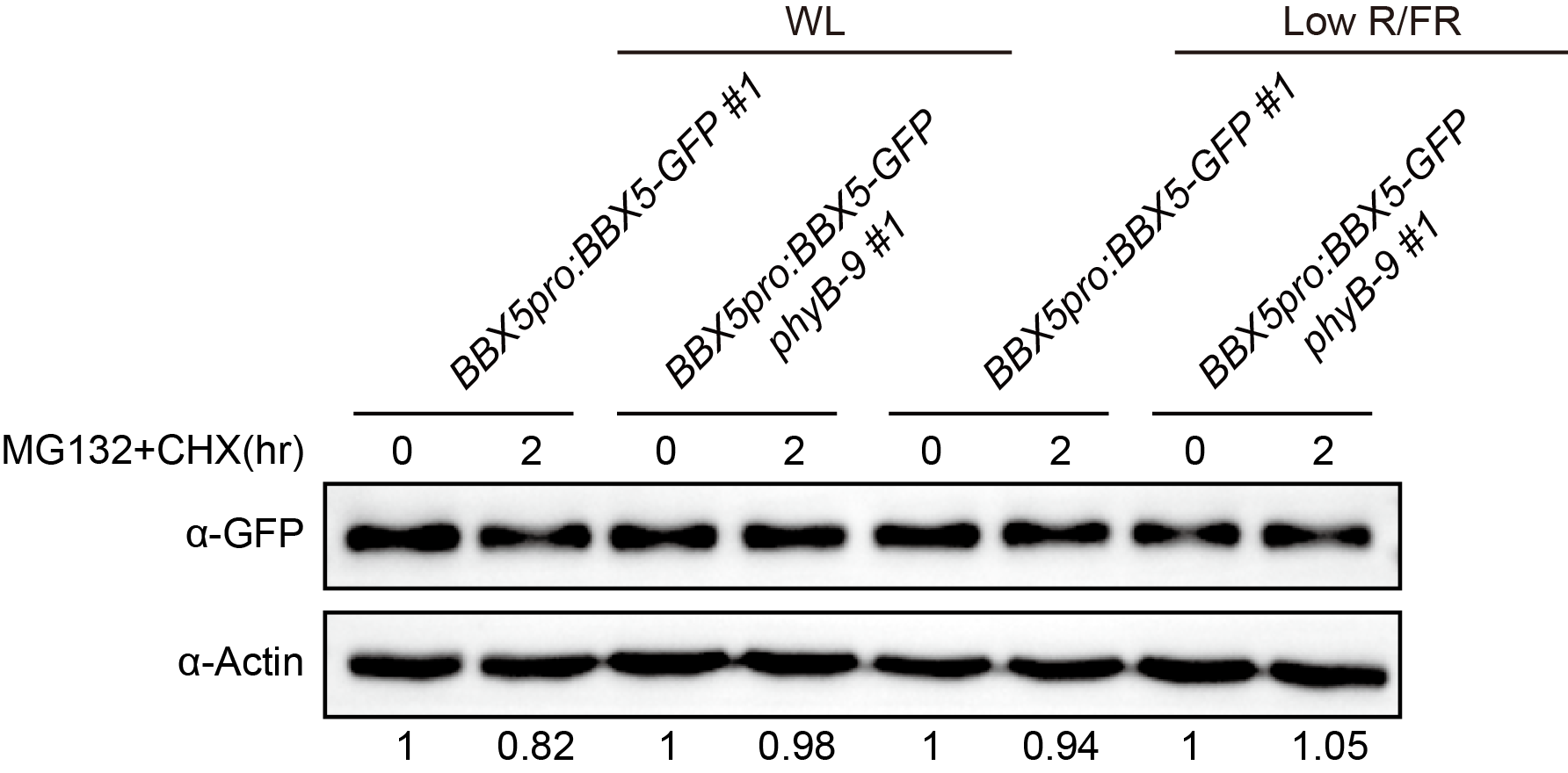

Supplement: S5 Fig — Immunoblots analysis showing BBX5-GFP protein levels in BBX5pro: BBX5-GFP #1 and BBX5pro: BBX5-GFP phyB-9 #1 seedlings after GM132 and CHX treatment. Plants were grown in WL for 5 d, then pretreated with MG132 (200 μM) for 3 h, and subsequently incubated with CHX (500 μM) for 2 h under WL or low R/FR conditions respectively. Actin was used as the loading control. Numbers below the immunoblots indicate the relative intensities of BBX5-GFP bands normalized to those of loading controls, and the ratio was set to 1 for the first lane of each seedling. (TIF) [file pgen.1012177.s005.tif]

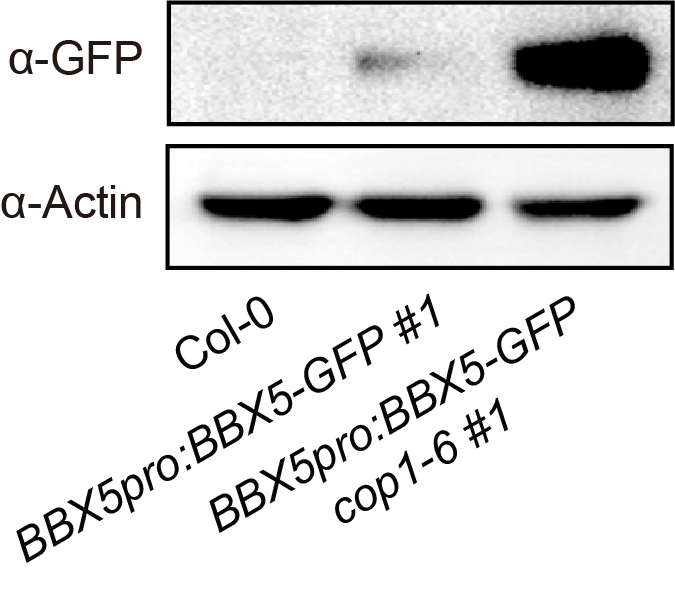

Supplement: S6 Fig — Immunoblots showing the BBX5-GFP protein levels in BBX5pro:BBX5-GFP #1 and BBX5pro:BBX5-GFP cop1–6 #1 seedlings. Plants were grown in the dark for 4 d. Col-0 was used as the negative control. Actin was used as the loading control. (TIF) [file pgen.1012177.s006.tif]

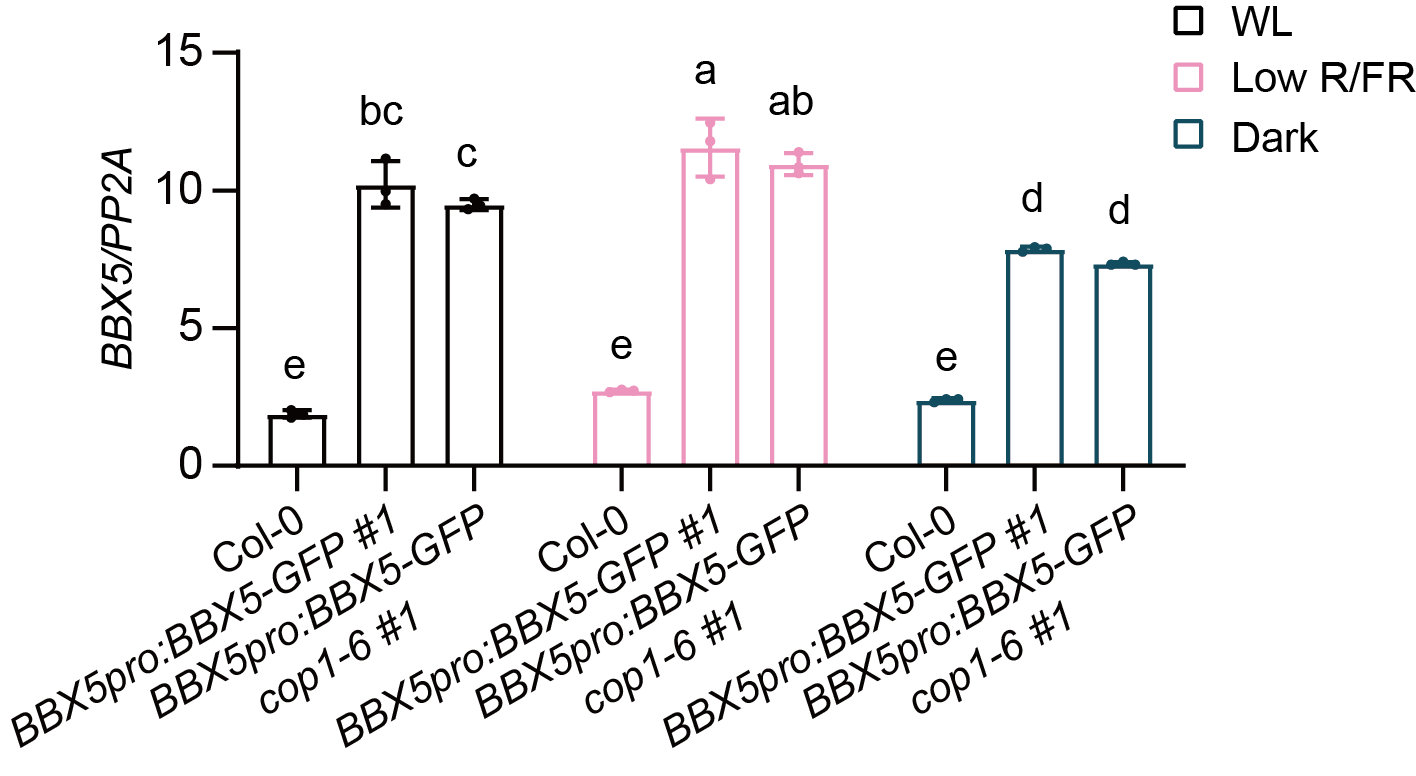

Supplement: S7 Fig — RT-qPCR analysis of BBX5 transcript levels in Col-0, BBX5pro:BBX5-GFP #1 and BBX5pro:BBX5-GFP cop1–6 #1 seedlings. Plants were grown in dark for 4 d, or grown in WL for 5 d and then either kept in WL or transferred to low R/FR for an additional 1 h respectively. Values are means ± SD (n = 3). Letters above the bars indicate significant differences (P < 0.05), as determined by one-way ANOVA with Tukey’s post hoc analysis. (TIF) [file pgen.1012177.s007.tif]

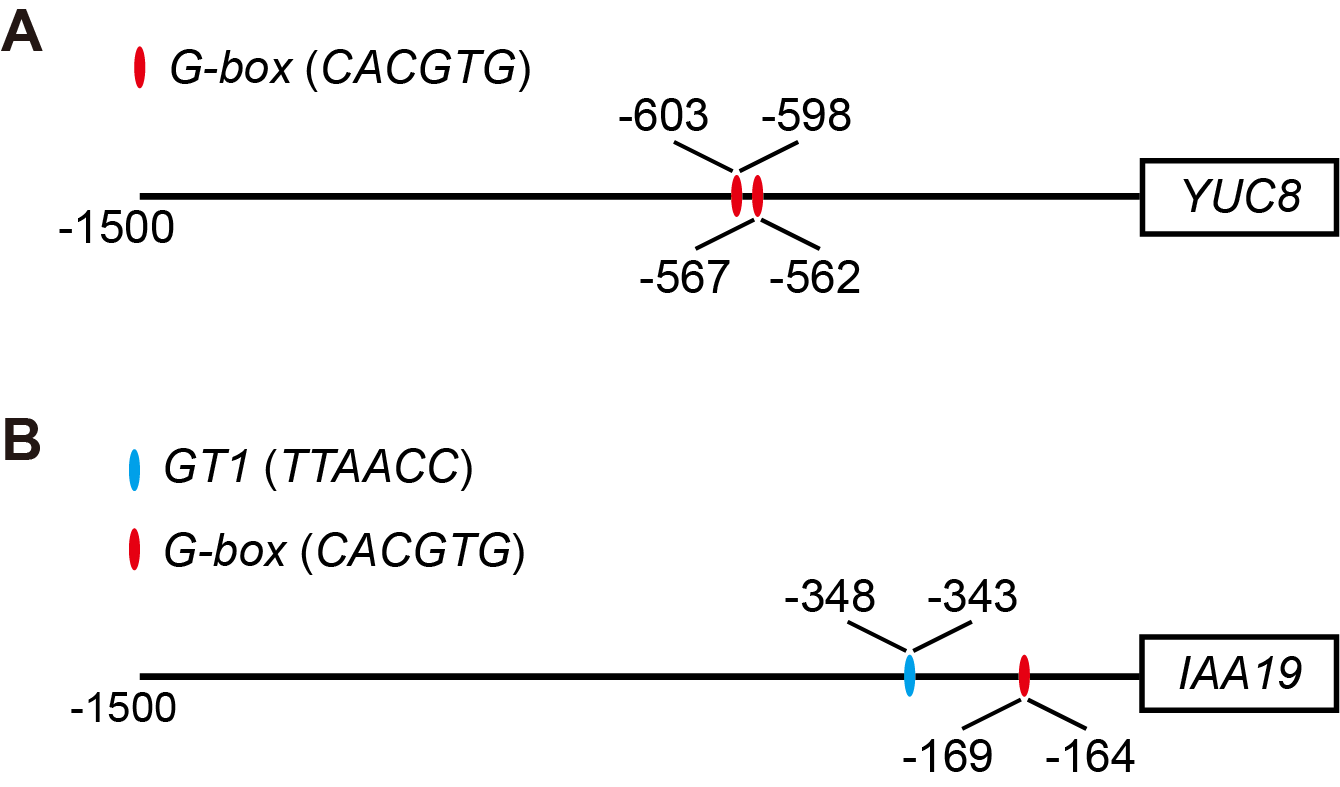

Supplement: S8 Fig — (A) Immunoblots showing the BBX5-Flag protein levels in 35Spro:BBX5-Flag transgenic seedlings. Plants were grown in continuous WL for 5 d. Col-0 was used as the negative control. Actin was used as the loading control. (B-C) Hypocotyl phenotypes (B) and length (C) of Col-0, bbx5–1 and 35Spro:BBX5-Flag seedlings. Plants were grown in WL for 3 d, then either kept in WL or transferred to low R/FR conditions for 3 d. Values are means ± SE (n ≥ 20). Scale bar = 1 mm. Letters above the bars indicate significant differences (P < 0.05), as determined by one-way ANOVA with Tukey’s post hoc analysis. (TIF) [file pgen.1012177.s008.tif]

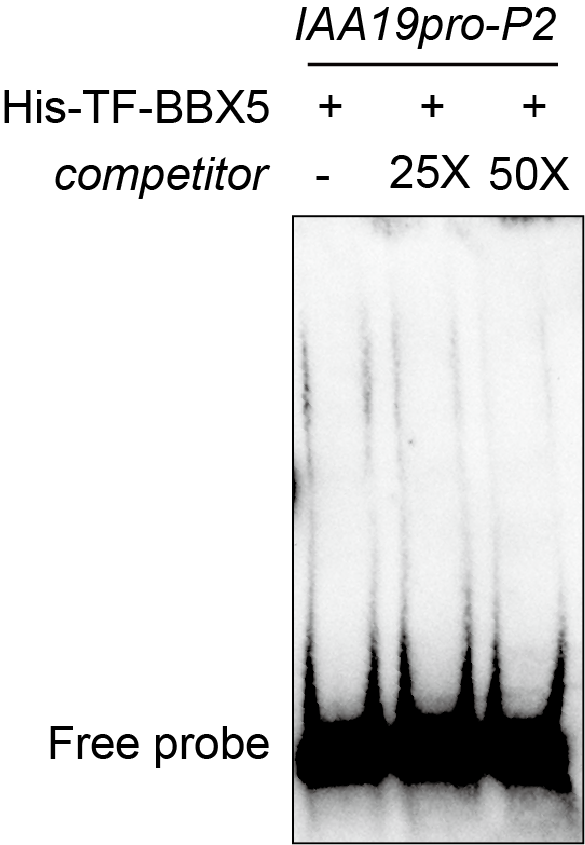

Supplement: S9 Fig — Schematic representation of the YUC8 and IAA19 promoter with the location of the G‐box and GT1-motif. The numbers indicate the positions of the G-box and GT1 motifs. (TIF) [file pgen.1012177.s009.tif]

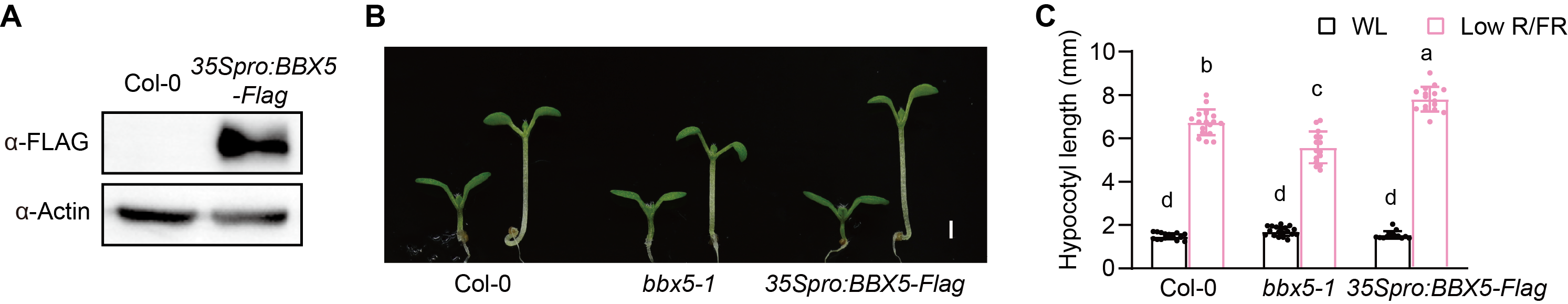

Supplement: S10 Fig — EMSA showing that BBX5 did not bind to the IAA19pro-P2 subfragments containing one G-box motif in vitro. The “+” and “−” indicate presence and absence, respectively. (TIF) [file pgen.1012177.s010.tif]
